# The developing xylem transcriptome and genome-wide analysis of alternative splicing in *Populus trichocarpa* (black cottonwood) populations

**DOI:** 10.1186/1471-2164-14-359

**Published:** 2013-05-29

**Authors:** Hua Bao, Eryang Li, Shawn D Mansfield, Quentin CB Cronk, Yousry A El-Kassaby, Carl J Douglas

**Affiliations:** 1 Department of Forest Sciences, University of British Columbia, Vancouver, BC V6T1Z4, Canada; 2 Department of Botany, University of British Columbia, Vancouver, BC V6T1Z4, Canada; 3 Department of Wood Science, University of British Columbia, Vancouver, BC V6T1Z4, Canada

## Abstract

**Background:**

Alternative splicing (AS) of genes is an efficient means of generating variation in protein structure and function. AS variation has been observed between tissues, cell types, and different treatments in non-woody plants such as *Arabidopsis thaliana* (*Arabidopsis*) and rice. However, little is known about AS patterns in wood-forming tissues and how much AS variation exists within plant populations.

**Results:**

Here we used high-throughput RNA sequencing to analyze the *Populus trichocarpa* (*P. trichocarpa*) xylem transcriptome in 20 individuals from different populations across much of its range in western North America. Deep transcriptome sequencing and mapping of reads to the *P. trichocarpa* reference genome identified a suite of xylem-expressed genes common to all accessions. Our analysis suggests that at least 36% of the xylem-expressed genes in *P. trichocarpa* are alternatively spliced. Extensive AS was observed in cell-wall biosynthesis related genes such as glycosyl transferases and C2H2 transcription factors. 27902 AS events were documented and most of these events were not conserved across individuals. Differences in isoform-specific read densities indicated that 7% and 13% of AS events showed significant differences between individuals within geographically separated southern and northern populations, a level that is in general agreement with AS variation in human populations.

**Conclusions:**

This genome-wide analysis of alternative splicing reveals high levels of AS in *P. trichocarpa* and extensive inter-individual AS variation. We provide the most comprehensive analysis of AS in *P. trichocarpa* to date, which will serve as a valuable resource for the plant community to study transcriptome complexity and AS regulation during wood formation.

## Background

Alternative splicing (AS) is considered to be a key factor underlying increased cellular and functional complexity in higher eukaryotes and has been studied extensively on the genome-wide scale in humans, other animals, and plants [1-5]. In humans, RNA splice variants with alternative exon configurations often accumulate differentially across different tissues and individuals [1,6-8] and such tissue-specific gene isoforms can have important functions in development and in the functioning of different cell types.

Relatively few studies have investigated genome-wide patterns of AS in plant species [9], but recent results from *Arabidopsis*[10] and rice [11] have revealed high levels of AS that can vary in different organs and under different stress conditions. Next generation high throughput transcriptome sequencing (RNA-Seq) analysis suggests that 42% of the intron-containing genes in *Arabidopsis* undergo AS [10]. Using a normalized cDNA library derived from flower and seedling tissue, Marquez et al. [5] used deep RNA-Seq transcriptome analysis to show that over 60% of *Arabidopsis* intron-containing genes are alternatively spliced, with intron retention (IR) being the most common form of AS. The analysis revealed, however, that over 50% of genes surveyed display non-IR AS, and within IR variants, a large number of cryptic introns were spliced out in-frame. Thus, as in humans, large scale AS in plants is likely to contribute to proteome and phenotypic diversity.

Few studies have addressed AS variation among individuals within a species, although it has been noted that extensive AS variation in humans may underlie phenotypic diversity and disease susceptibility [7], and mutations affecting alternative splicing can play a role in disease [12]. Recently, RNA-Seq was used for genome-wide analysis of variation in AS splicing and expression levels across human individuals [13,14]. High depth transcriptome sequencing of cell lines derived from two unrelated individuals revealed a large number of genes differentially spliced between the two cell lines, and many of these differences were associated with single nucleotide polymorphisms (SNPs) found in close proximity [13]. This and other studies [14] analyzing expression level variation and AS variation in individuals from the human HapMap project suggested that genetic variation (e.g., SNPs) underlies extensive AS and expression level variation among unrelated individuals, which appears to contribute to phenotypic diversity. However, variation of alternative splicing within plant population has not yet been subject to genome-wide analysis.

The sequencing of the first tree, *Populus trichocarpa * (black cottonwood; referred to as “poplar” throughout) [15], creates opportunities for genomic studies in this species, and in particular for investigation of biological processes important in woody plants, such as perenniality, secondary growth, and secondary xylem (wood) development [16]–[18]. *P. trichocarpa* is the largest deciduous tree native to western North America with a range that extends from California to Alaska, USA, a latitudinal range of over 30°. One advantage of woody plants such as *P. trichocarpa* and other trees is the ability to isolate pure tissue types, i.e. developing xylem and phloem, from woody stems during the active growth period, which can be used for studies of tissue-specific transcriptomes and proteomes. Published studies on the poplar xylem transcriptome, and the xylem transcriptomes of other woody plants, have been largely limited to use of EST and full-length cDNA resources from traditional sequencing platforms [19-23]. Furthermore, the annotation of AS forms in the 40,668 loci containing protein-coding transcripts a recent annotation of the *P. trichocarpa* genome (version 2.2; http://www.phytozome.net/poplar) is limited. Given its large population size across a large geographical range of varied environments, and extensive genetic polymorphism [24,25], study of *P. trichocarpa* transcriptomes offers the opportunity for the systematic identification and characterization of splice variants at the population level, which will be of critical importance for understanding phenotypic variation in wood and other traits in the these populations.

The advent of next-generation sequencing technologies has now enabled AS analyses at unprecedented levels of sensitivity and precision. Analysis of Illumina-based RNA sequencing (RNA-Seq) data has been particularly useful in the study of AS and AS variation in model species [1,3,5,10]. We recently used Illumina paired-end sequencing of xylem expressed transcripts in 20 individuals from populations (12 from southern and 8 from northern populations) across much of the *P. trichocarpa* species range to gain initial insights into the poplar xylem transcriptome [24]. This analysis generated an average depth of sequence coverage of >48 X across over 18,000 xylem-expressed genes, and revealed extensive single nucleotide polymorphism (SNP) variation, with over 500,000 putative SNPs identified [24]. This work provided a rich data set for further investigation of the poplar xylem transcriptome, including expression levels and AS.

Here, we report the use of the *P. trichocarpa* developing xylem RNA-Seq data set [24] to investigate xylem gene expression, to query the extent and complexity of AS in *P. trichocarpa*, and to investigate potential AS variation among different individuals from different populations. Our data provide an unprecedented and unbiased evaluation of alternative splicing in this species. The RNA-Seq data confirmed most annotated splice junctions and identified tens of thousands of novel AS events. We found that 36% of the *P. trichocarpa* xylem-expressed genes are alternatively spliced. This estimate is significantly higher than previous estimates based on cDNA/EST sequencing [26]. Additionally, we quantified splice variant expression levels (isoform frequency) across all 20 individuals. A large number of AS events were identified that had markedly different isoform ratio in individuals within the studied two geographically separated sets of populations.

## Results

### Mapping of the *P. trichocarpa* developing xylem transcriptome

**Figure 1:**
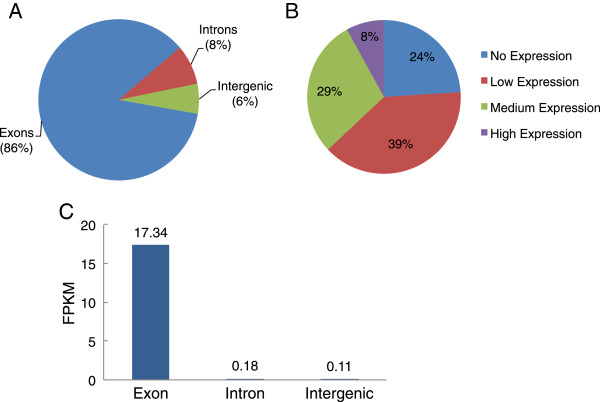
**Mapping of Illumina reads to the *P. trichocarpa *genome. **(**A**). Distribution of Illumina GAII RNA-Seq reads along annotated poplar annotated genomic features (*P. trichocarpa* v. 2.2). (**B**). Summary of the average expression level across 20 individuals. (High: FPKM>40, Medium: FPKM >5-40, Low: FPKM 0–5) (**C**). Expression level of exon, intergenic, intron. Mean read density values for exons, introns, and intergenic regions were computed in units of fragments per kilobase of exon/intron/intergenic (FPKM) model.

Twenty *P. trichocarpa* individuals from a population of approximately 450 individuals from the BC Ministry of Forests collection [27,28] maintained in a common garden at the University of British Columbia were selected for xylem transcriptome analysis using the Illumina platform for ultrahigh-throughput RNA sequencing (RNA-Seq). The origins of the selected individuals range from 59.6°N to 44.0°N (Additional file 1), including 12 individuals (PT02-PT13) from southern populations (~44°- 54°N) and 8 (PT14-PT21) from northern populations (~58°- 60°N). We acquired a total of more than 1,660 million paired-end reads of 36-50 bp in length for xylem of the studied 20 individuals (Additional file 2). All short reads were aligned onto the reference *P. trichocarpa* genome v. 2.2 at single bp resolution using SOAP software [29]. In total, 63.6% of the RNA-Seq reads were uniquely mapped onto the reference genome (Additional file 2) and, as expected, most of these (86%) mapped to annotated exons (Figure 1A). However, a significant fraction mapped to sequences annotated only as introns (8%) and to regions without annotated genes (intergenic; 6%) (Figure 1A). We did not investigate in detail the nature of the reads that mapped uniquely to intergenic regions, but preliminary analyses suggest that many are small, highly expressed transcriptional units, some with open reading frames, that are not obviously associated with annotated genes (i.e., non-annotated exons of known genes) (N. Farzaneh, A. Geraldes, and C. Douglas, unpublished).

To explore potential AS events, we used SOAPsplice [30] to discover splice junctions (see Methods). 8.7% of all reads were aligned on splice junctions (Additional file 2). Overall, using the total xylem transcriptome data from 20 individuals, we detected 62% (118,751) of known junctions in the *P. trichocarpa* genome. In addition, we identified 80,345 new splice junctions not in the *P. trichocarpa* genome annotation (see Additional file 3 for the number of known and new splice junctions detected in each individual). This indicates a relatively high number of AS variants for which no previous experimental data exists.

It is now clear that measurements of gene expression levels from RNA-Seq are correlated with measures of absolute expression level (as assayed by microarray) across a wide dynamic range [31]. We quantified the expression levels of all genes in our RNA-Seq data set by determining the number of fragments per kilobase of exon in a gene per million fragments mapped (FPKM; [32]). We investigated the expression levels (FPKM value) of all 40,668 annotated genes (*P. trichocarpa* v. 2.2) across 20 individuals and found that 24% of genes per individual had no coverage (FPKM=0, or no reads mapped) (Figure 1B). We observed high (FPKM >40) expression for an average 8% of the genes per individual. Medium (FPKM ≥5 to ≤ 40) and low (FPKM >0 to ≤ 5) expression levels were observed for 29 and 39% of the genes, respectively. The depth of read matches to intergenic regions and annotated gene features is illustrated in Figure 1C. As expected, the depth of coverage of annotated intergenic regions and introns was much lower than that for exonic features.

Several highly expressed genes (see Additional file 4 for the the complete data set of FPKM values for 41,377 gene models in all 20 individuals, and for the 100 most highly expressed genes in 20 individuals) encode proteins involved in secondary cell wall biosynthesis, including lignin biosynthesis (caffeic acid *O-*methyltransferase, COMT; caffeoyl CoA 3*-O-*methyltransferase, CCoAOMT; cinnamyl alcohol dehydrogenase, CAD; cinnamate 4-hydroxylase C4H; ferulate, F5H; coumarate 3-hydroxylase C3′H; hydroxycinnamoyl CoA:shikimate/quinate hydroxycinnamoyltransferase; HCT; Hamberger et al., 2007), cellulose biosynthesis (Chitinase-like 2, CTL2; KORRIGAN), and glucuronoxylan biosynthesis (glucuronosyltransferase, IRX10). Additionally, genes encoding enzymes in methionine metabolism (homocysteine S-methyltransferases and methionine adenosyltransferases), cytoskeleton components (TUB, TUA, ACT), sucrose synthase (PtrSuSY1, PtrSuSY4), and encoding several AGP/FLA arabinogalactan proteins that have been implicated in secondary wall biosynthesis are among the most highly expressed genes. This is consistent with the strong metabolic commitment of this tissue to secondary wall biosynthesis. Interesting, among the 100 most highly expressed genes, over 10% (12) encode proteins of unknown function, and 8 of these have no obvious *Arabidopsis* homologs, and are poorly conserved in other plants (http://www.phytozome.net). The most highly expressed gene encoding a protein of unknown function is POPTR_0002s22040 (FPKM 1178), which has no homologs in other plants except cassava, which is related to *P. trichocarpa*. This suggests that many unknown functions potentially contributing to xylem development and secondary cell wall biosynthesis remain to be discovered.

### Identification of alternative splicing in the xylem transcriptome

**Figure 2:**
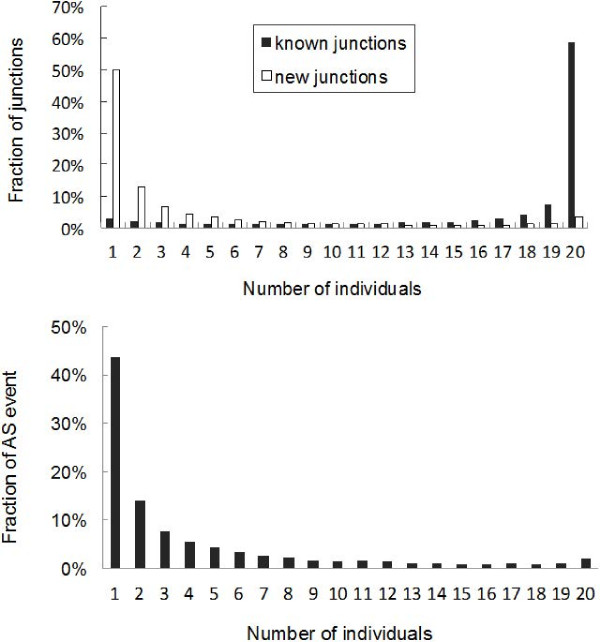
**Distribution of junctions and alternative splicing events **(**A**). Histograms of the distribution of known and new splice junctions among 20 *P. trichocarpa* individuals. We required splice junctions to be supported by at least two reads with a non-repetitive match position and with a minimum of four bases on both sides of the junction. (**B**). Histograms of the distribution of alternative splicing events among 20 individuals. Only genes with sufficient read coverage (FPKM ≥5) in all individuals were used.

A prerequisite for a comprehensive survey of alternative splicing is the ability to reliably detect splice junctions. Our global survey of transcript variants based on the 20 poplar xylem transcriptomes identified 199,096 splice junctions, 118,751 of which were known junctions and 80,345 were new junctions absent in the annotated genome (Additional file 3). We observed, on average for one individual, 3.4 junctions per gene and a mean density of 41.8 reads per junction. To assess the degree to which known and new junctions detected in the mRNA-Seq data may represent AS events that vary between individuals, we investigated the frequencies at which splice junctions were unique to one individual, or were found in common to two or more individuals. Most known junctions were detected in nearly all individuals (Figure 2A). However, among the new junctions revealed in our RNA-Seq data, a significant number (50%) were detected in only one individual, and few were detected in all or most individuals (Figure 2A and B). These could represent genotype-specific AS variants. It is also possible that they include lower-abundance splice variants that are widely expressed but were only detected in a single or few individuals and/or partially processed transcripts that were only detected in a single or few individuals.

**Figure 3:**
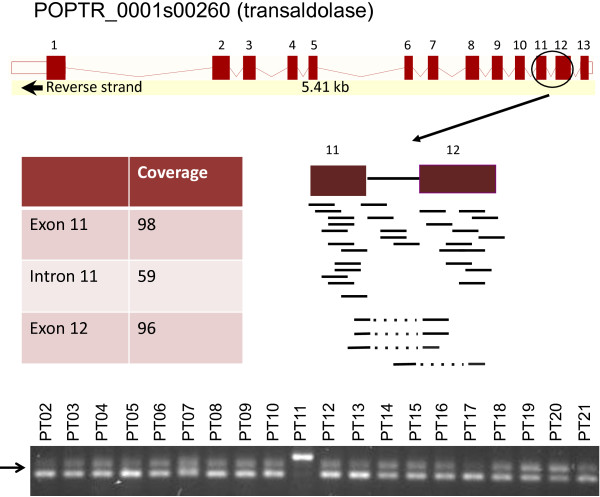
**Example of AS event observed by RNA-seq and RT-PCR. **Top, gene model for Poptr_0001s00260 (annotated as encoding a transaldolase) from the Phyotzome browser of *P. trichocarpa *genome, v. 2.2 (http://www.phytozome.net). Exons are numbered 1–13. The 11th intron is retained in a splice variant. The average coverage is shown for the retained intron and adjacent exons in sample PT19. The mapped reads are presented below the illustrated retained intron and adjacent exons by short black lines, and the junction reads are denoted by the broken reads below the gene structure models. Bottom, RT-PCR analysis of Poptr_0001s00260 transcripts in RNA samples from individual poplar trees. Arrow indicates longer RNA isoform due to intron retention.

To explore potential AS events, adjacent exons were joined into multi-exon genes via the spliced junctions. We empirically classified the AS events into four different types according to the structures of exons (see Methods). An illustrative example is shown for POPTR_0001s00260, annotated as encoding a transaldolase, in Figure 3. The sequence coverage and junction reads clearly reflect an intron retention AS event at intron 11 (Figure 3). To validate this result, we used RT-PCR on xylem RNA from each of the 20 individuals with RNA-Seq data. Evidence for the predicted intron retention isoform was found in all 20 individuals tested, at abundances consistent with RNAseq read counts.

For each individual, 7-11% of the total (40,668) annotated genes showed evidence for AS. In total, 11,232 *P. trichocarpa* xylem-expressed genes had evidence of AS, for a total of 27,902 AS events (Tables 1 and 2; Additional file 5). These events were categorized into the four known types of AS models, as shown in Table 2. In our data, we found that 76% of all annotated genes had some level of sequence coverage in xylem. Therefore, in total, we estimate that at least 36% of xylem-expressed genes are alternatively spliced. Recent evidence based on next-generation sequencing indicates a high incidence of AS in the human (86% of annotated genes), *Arabidopsis* (60%) and rice (33%) genomes [1,10,11]. We found that intron retention is the most prevalent form of alternative splicing (40% of the AS events), while exon skipping only constituted 8% and alternative 3′ and 5′ splice sites were at intermediate frequencies (Tables 1 and 2). This is in contrast to mammals and human where exon skipping is the most prevalent mechanism. Thus, based on previous analyses in plants, and now in *P. trichocarpa*, intron retention seems to be the most common AS feature in plants.

**Table 1:** Number of AS genes and events in 20 individuals

**Individual**	**AS gene**	**A3SS**	**A5SS**	**IR**	**ES**
PT02	2972	1209	708	1870	257
PT03	2843	1082	595	2032	227
PT04	4047	1796	1003	3115	358
PT05	3200	1481	837	1782	321
PT06	2950	1340	767	1868	278
PT07	3658	1469	795	3120	331
PT08	3751	1510	894	3074	285
PT09	3833	1548	938	3124	299
PT10	2915	1423	815	1446	247
PT11	2357	893	484	1522	174
PT12	3663	1832	1048	1991	236
PT13	3750	1796	1041	2093	325
PT14	4088	1614	952	3176	488
PT15	4584	2059	1305	3232	416
PT16	3470	1720	1044	1647	306
PT17	4422	1799	1102	3301	388
PT18	4346	2382	1464	2517	336
PT19	4567	2145	1299	3131	451
PT20	4478	2471	1498	2737	355
PT21	3510	1807	1161	1500	402
Total	11232	8963	5545	11175	2219
Fraction^1^	27%	32%	20%	40%	8%

^1^Fraction of AS genes: 11232 divided by number of annotated genes (40,668). Fraction of AS events: number of each type AS events.

**Table 2:** Summary of alternative splice events detected by RNA-Seq

	**Alternative splice (AS) type**^**1, 2**^				
	**A3SS**	**A5SS**	**IR**	**ES**	**Total**
Number AS events detected	8963	5545	11175	2219	27902
% of AS events	32.1	19.9	40.0	8.0	100
Number in 1 individual	4560	2873	4194	1224	12851
Number in ≥ 15 individuals	572	310	651	59	1592
Number in 20 individuals	195	80	134	15	424
% in 1 individual only	50.1	51.2	37.5	55	46
% in ≥15 individuals	6.4	5.6	5.8	2.7	5.7
% in 20 individuals	2.2	1.4	1.2	0.7	1.5

^1^A3SS, alternative 3′ splice site; A5SS, alternative 5′ splice site; IR, intron retention; ES, exon skip.

^2^Data are for all AS events detected.

We next analyzed to what extent AS events observed in one individual were also observed the other 19 individuals. Table 2 shows that the bulk of the AS variants were observed in one or a few individuals. To exclude low abundance transcripts that could represent “transcriptional noise” of biological or technical origin, the significance of which is under debate [33], we filtered the data to include only splice variants of genes with FPKM values of ≥ 5 in all individuals. As shown in Figure 2B, around 46% of AS events in this data set were individual-specific, and only 2% were conserved in all 20 individuals. Since the developing xylem tissue from individuals of the southern (PT02-PT13) populations and northern (PT14-PT21) populations were harvested in two different years (from the same common garden, same week, and same time of day), the distribution of AS events for these two experiments were analyzed separately, but the distributions were essentially the same as the combined analysis shown in Figure 2. This indicates a high degree of alternative splicing polymorphism within this species that would not be detected if only a single or few individuals had been sampled.

*P. trichocarpa* is considered a model system for the study of wood (secondary xylem) development [34], which involves differentiation of vessel element and fiber cells from derivatives of the vascular cambium stem cell population. These cells undergo cell expansion followed massive secondary wall deposition and lignification, and programmed cell death [35]. Many of the genes involved in secondary wall formation, and transcriptional regulators of this process [36] are well known. To investigate the pattern of AS in these genes, we generated a dataset of 389 cell-wall biosynthesis related and 598 xylem-expressed transcription factor (TF) genes (Additional file 6). We found that 38.9% (149) of the cell-wall biosynthesis related genes have evidence for alternatively splicing, for a total of 424 AS events. Of the transcription factor genes, 38.7% (232) showed evidence of alternative splicing, for a total of 584 AS events.

An important class of secondary cell wall related enzymes are glycosyl transferases, and we found evidence for extensive alternative splicing of glycosyl transferase genes (with 130 events in 29 genes). For example, POPTR_0005s06280, a glycosyl transferase highly expressed in developing xylem (FPKM=182) and homologous to the *Arabidopsis GLUCURONIC ACID SUBSTITUTION OF XYLAN4* (*GUX4*) gene involved in xylan biosynthesis [37], exhibited 16 AS events across the 20 individuals, with 2 events common to ≥ 15 individuals and 3 unique to a single individual (Additional file 6). POPTR_0007s04030, similar to *Arabidopsis GUX1*, a second *GUX* gene highly expressed in developing xylem (FPKM =141), exhibited three AS slice forms common to all 20 individuals: two alternative 3′ splice acceptor sites in the second intron, and an alternative 5′ splice acceptor site in the fourth intron (Additional file 6). These data suggest that the diversity of glycosyl transferase proteins involved in secondary cell wall polysaccharide biosynthesis could be greatly expanded by AS in developing secondary xylem. It is interesting to note that a particular feature of human glycosyl transferases is alternative splicing, which leads to variability in the N-terminal regions of the proteins [38].

Among the TF genes, we found evidence for AS in members of the all TF classes examined (Additional file 6). Among these events, 42 were common to ≥ 15 individuals, while 242 (41%) were unique to one individual. We found 15 AS events in 12 TF genes that were common to all individuals, including events in homologs of *Arabidopsis ANAC078/NAC2* (POPTR_0010s23650), *KNAT6* (POPTR_0012s08910), and MYB103 (POPTR_0003s13190), a gene implicated in regulation of cell wall biosynthesis [39]. Interestingly, two duplicated genes encoding C2H2 zinc transcription factors (POPTR_0003s07180 and POPTR_0001s16080, both homologs of the *Arabidopsis SUPPRESSOR OF FRIGIDA4* (*SUF4*) C2H2 gene, had intron retention AS forms in all 20 individuals. The *SUF4* gene is alternatively spliced in *Arabidopsis*[40], and a similar AS pattern, retention of the last intron, is found in both the poplar homologs and in the *Arabidopsis SUF4.3* splice variant. All cell wall formation and TF related genes with AS variants are listed in Additional file 6.

### Alternative splicing isoform variation between individuals

Although most documented AS isoform variation occurs between tissues or environmental conditions, differences exist among individuals [12-14]. In addition to qualitatively detecting AS events, Illumina-based RNA-Seq can generate reliable quantitative measurements of AS levels. For each of AS event type, reads derived from specific regions incorporated into an mRNA indicate quantitative expression of one alternative RNA isoforms. We used an inclusion and exclusion of junction reads method to quantify isoform expression in the RNA-Seq data set (see Methods).

**Figure 4:**
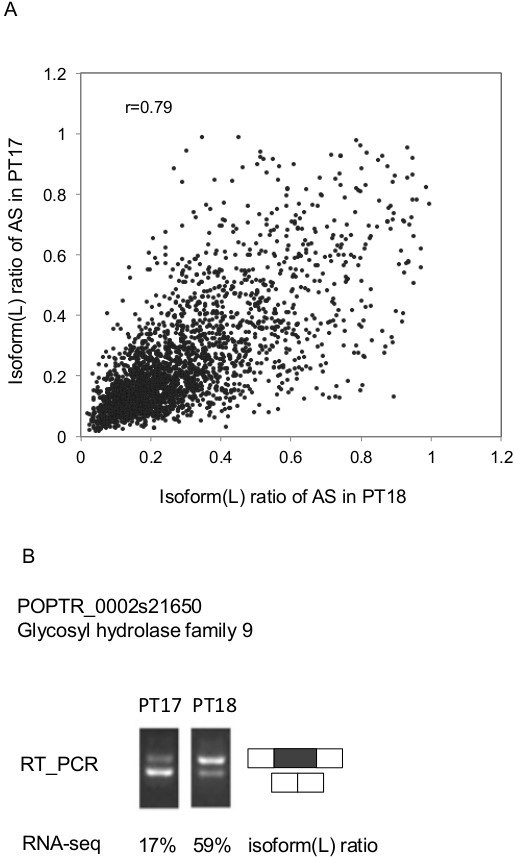
**Differences in isoform frequency between two individuals. **(**A**). Scatter plot showing all long isoform (intron retention) AS frequencies between two individuals (PT17 and PT18). Each point represents an intron retention event. (**B**). An example of significant isoform variation in RNA specific to Poptr_0002s21650 (predicted to encode a glycoside hydrolases family 9 protein) between PT17 and PT18 and RT-PCR validation of Poptr_0002s21650 isoform frequency variation in PT17 and PT18. The migration of PCR products corresponding the long (L, intron retention) and short (S) isoforms by gel electrophoresis is shown, with the relative frequencies was estimated by RNA-Seq data.

Using this approach to compare the isoform ratio in each individual relative to the other individuals, classes of AS events were observed that had markedly different isoform ratios between different individuals. One example is shown in Figure 4A, which depicts a scatterplot of all long (inclusion) isoform ratios of events in individual PT17 against individual PT18 both from the north populations. Although there was an overall good correlation between isoform (L) ratios in the two individuals (r = 0.79), many outliers were evident. One such event was in POPTR_0002s21650 (predicted glycosyl hydrolase family 9 protein/ endo-1,4-beta-glucanase). This gene contains 4 exons and the first intron can be either retained or spliced out. Intron retention is the dominant isoform in PT18 but the minor isoform in PT17. RNA-Seq data indicated significantly different ratios of the L (intron retention) vs. short (S; intron spliced) in the two individuals (Figure 4B). This result was validated by RT-PCR (Figure 4B).

**Figure 5:**
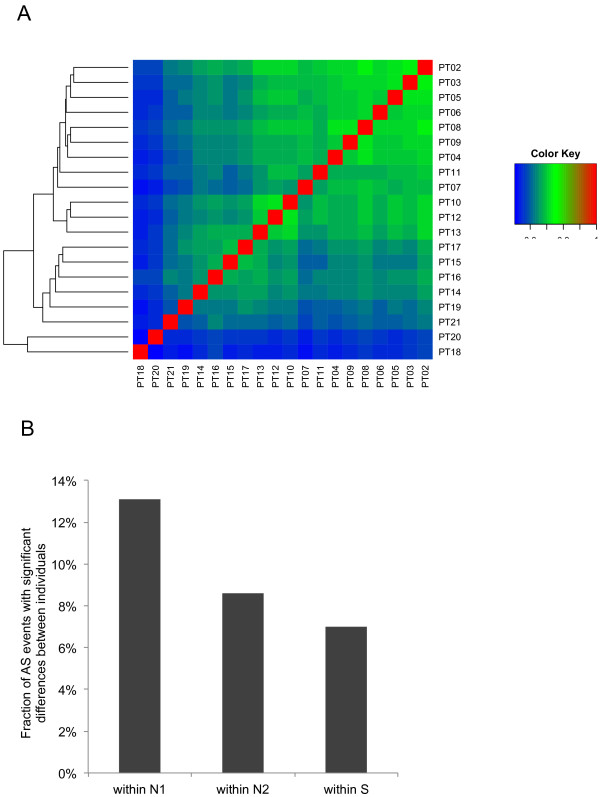
**The extent of alternative isoform expression differences within population sets.** (**A**). Spearman correlations of isoform (L) ratios between individuals. Correlations were computed separately for each pair of individuals, and clustered according to similarity using average linkage hierarchical clustering. Only genes with sufficient read coverage (FPKM ≥5) in all individuals were used. (**B**). The fractions of AS events show significant differences between at least two individuals within population sets (N1, 8 individuals from northern populations; N2, 6 individuals (excluding more divergent PT18 and PT20 samples) from northern populations; S, 12 individuals from southern populations). Variation in AS events at an adjusted Fisher’s exact *P-*value cutoff corresponding to a false discovery rate of 5% between at least one pair of individuals within the population set were considered significant. Only genes with sufficient read coverage (FPKM ≥5) in all individuals were used.

For the RNA-Seq dataset we sampled developing xylem from 12 individuals taken from the southern area of the *P. trichocarpa* collection, and 8 from the northern area of the collection (Additional file 1) that were grown in a common garden. To assess AS isoform variation between *P. trichocarpa* individuals in these populations on a global level, the correlations among the vectors of L-isoform frequencies for all detected AS events between pairs of samples were determined (Figure 5A). To control different sequence coverage of different individuals, we also filtered the data to include only splice variants of genes with FPKM values of ≥ 5 in all individuals. Correlations were clustered according to similarity using average linkage hierarchical clustering. Samples from individuals in north populations had much more variation than samples from those in the southern populations, and overall, Spearman rank correlations (r) ranged from of 0.75 to 0.87, indicating that the level of variation observed in the PT17 vs. PT18 comparison is common between individuals sampled. One striking observation in this analysis was the strong clustering of the 12 individuals from southern populations (PT02-PT13) by their frequency of shared isoforms. Pairs from southern population had generally higher correlations between each other than with individuals from the northern populations (PT14-PT21) and hierarchical clustering based on these results identified two groups that correspond to the northern and southern populations (Figure 5A). This suggests that xylem RNA samples isolated from the northern populations are more heterogeneous in their AS variation than those isolated from the southern populations. Two samples, PT18 and PT20, are from northern population individuals within close geographical proximity, yet show the lowest AS similarity. We have recently observed that *P. trichocarpa* individuals from northern populations exhibit higher nucleotide diversity than those from southern populations (A. Geraldes, C. Douglas, and Q.C. Cronk, unpublished), and these trees grow in a region in which hybridization with closely related species *P. balsamifera* may occur. Thus, one explanation for this finding is that introgression of alleles from P*. balsamifera* into one or both of these two individuals has led to higher than expected genetic diversity, reflected in the observed lower than expected isoform correlation. Alternatively, environmentally induced physiological variation could have affected AS variation differently in the northern individuals in the year the xylem samples were isolated.

Next, we investigated how many AS events showed significant difference between individuals within and among the population. To control the different sequencing coverage of different individuals, we filtered the data to include only splice variants of genes with FPKM values of ≥ 5 in all individuals. We found 7.0% and 13.1% (8.6%, excluding more divergent PT18 and PT20 samples) of AS events respectively that are predicted to have significant differences (FDR< 0.05) in the isoform ratio between at least two individual pairs within southern and northern populations (Figure 5B). This was also consistent with the lower correlation of isoform ratios in north populations relative to southern populations (Figure 5A). We are not aware of similar analyses in plants, but they are in general agreement with an analysis of EST and cDNA data, which estimate that 21% of human AS genes are affected by polymorphisms that alter the relative abundances of alternative isoforms [41], and with RNA-Seq analysis of 6 human individuals that estimate that between 10% and 30% AS events show individual-specific variation [1]. However, these frequencies are still below the level of ~60% variation in AS events among different human tissues [1]. This observation is not surprising, because the same tissues of different individuals are functionally more similar than different tissues of the same individuals.

## Discussion

Traditionally, genome-wide studies of AS were carried by sequencing of ESTs and using exon arrays, but such approaches were limited by cost and ability to only efficiently identify common AS events [42]. It is now feasible to conduct deep and comprehensive sequencing of transcriptomes in a high throughput and cost effective manner using next generation sequencing such as the Illumina platform employed in this study, making it possible to comprehensively survey both gene expression levels and splicing variation, and to detect rare AS events. In this paper we present a global analysis of gene expression and alternative splicing in the model tree species *P. trichocarpa*, focusing on over 30,000 genes expressed in the developing xylem tissue taken from twenty unrelated individuals. We compiled a catalog of xylem-expressed genes, including nearly 13,000 genes expressed at moderate to high levels (average FPKM ≥5; and 100 genes very highly expressed genes (FPKM > 460; Additional file 4). Using the RNA-Seq approach, we mapped and annotated the intron-exon junctions in these genes, which allowed us to assess splicing patterns on a global level in the twenty individuals analyzed.

A striking finding in our analysis, and one that demonstrates the power of next generation RNA-Seq to efficiently reveal diverse patterns of AS, is the observation that there are significantly more new (non-annotated in the *P. trichocarpa* reference genome) intron-exon junctions in the set of 20 unrelated individuals than were detected in any one single individual. Many of these may represent genotype-dependent AS variants and suggest a remarkable diversity in AS patterns. The bulk of the AS and AS variation we observed is likely to be biologically relevant. Similar levels of AS variation and isoform abundance variation were observed in two separately harvested sets of developing xylem (those from the 12 southern and 8 northern populations). Furthermore, selected examples of AS were verified by RT-PCR, many of the events are in moderately to highly expressed genes, many transcript variants were present at high levels, and we found examples of AS in *P. trichocarpa* genes known to have splice variants in *Arabidopsis* (see below). As RNA-Seq is quantitative, it can be used to accurately determine isoform expression levels that result from AS. In principle, it is possible to determine the absolute quantity of every RNA variant in a cell population, and directly compare results between experiments. Thus, in addition to revealing important new insights into alternative splicing complexity in the *P. trichocarpa* transcriptome, our results show that RNA-Seq data can be used to reliably measure AS levels in large sample sizes.

While AS in humans is common and up to 86% of genes have evidence of AS [1], AS in plants has not been as extensively studied. Furthermore, relatively few plant AS events have been functionally characterized, but evidence suggests that AS participates in important plant functions, including stress responses, and may impact domestication and trait selection. Recent evidence based on next-generation sequencing indicates a high incidence of AS *Arabidopsis* (35% - 60%) and rice (33%) genomes [1,5,10,11]. Consistent with those levels, our findings suggest that 36% of xylem-expressed transcripts in *P. trichocarpa* are alternatively spliced. This estimate of AS levels in *P. trichocarpa* is much higher than previous estimates based on cDNA/EST sequencing [26]. As well, only 3,063 out of 40,668 genes (7%) in the *P. trichocarpa* genome (v2.2) are annotated as having at least two transcripts. It is likely that the number of alternatively spliced genes identified in *P. trichocarpa* will increase with larger and more comprehensively sampled tissue-specific transcriptome sequence collections. In addition, plants respond to the environment in diverse and complex ways, and only a small proportion of these conditions have been addressed in next-generation sequencing projects that would reveal AS.

Until now, most studies of splicing variation have been in mammals and humans. Our study adds to the plant literature that has so far focused primarily on *Arabidopsis* and rice, which has suggested that plants and animals differ in the predominant AS types. As in *Arabidopsis*[10] and rice [11], we found that intron retention is the most prevalent form of alternative splicing accounting for 40% of the AS events (Tables 1 and 2). In contrast to humans where it is the predominant form, exon skipping only constituted 8% of the *P. trichocarpa* developing xylem AS events, consistent with results in *Arabidopsis* and rice. While recent work in *Arabidopsis* indicates that over 50% of genes display the non-IR AS [5], the relative prevalence of intron retention AS and paucity of exon skipping in all three plant taxa studied in depth (*Arabidopsis*, rice, *P. trichocarpa*) suggests that the mechanisms of splice site recognition and splice site selection differ between plants and animals.

We found many genes involved in cell wall biosynthesis and transcriptional regulation, which play key roles in secondary xylem development, are alternatively spliced. Future studies are needed to characterize changes in AS over the course of secondary xylem development, and the potential functional consequences of the AS observed. One way of assessing functional significance is conservation of AS across different taxa. While there are too few documented examples to make global comparisons, it is interesting to note that our initial analysis showed that two duplicated *P. trichocarpa* xylem expressed genes encoding C2H2 zinc transcription factors (POPTR_0003s07180 and POPTR_0001s16080) share a similar AS pattern with the *Arabidopsis SUPPRESSOR OF FRIGIDA4* (*SUF4*) C2H2 orthologous gene [40] in all 20 *P. trichocarpa* individuals, retention of the last intron as found in the *Arabidopsis SUF4.3* splice variant. Alternative splicing of *INDERMINATE DOMAIN 14* (*IDD 14*), which encodes another C2H2 transcription factor in *Arabidopsis* and rice, has been shown to be functionally important in a generating functionally distinct TF heterodimer that acts as a competitive inhibitor in regulating starch metabolism [43]. Conservation of alternatively spliced of genes of this class in *P. trichocarpa* and *Arabidopsis* and such functional information suggests that these splice variants may play biologically significant roles. Another example of the functional consequences of AS in *Arabidopsis* involves the modulation of the relative amounts of the peroxisomal versus cytosolic transthyretin-like (TTL) protein by AS, which affects ureide biosynthesis [44]. Given the metabolic and developmental complexity of secondary xylem and secondary wall formation, it would not be surprising if a number of the numerous AS events we observed in the mRNA population of this tissue were involved in developmental and metabolic regulation. Future studies in *P. trichocarpa* and other woody plants must focus on the functional consequence of AS on proteome diversity related to wood formation.

We found that 7% and 13% of AS events in developing xylem showed significant differences in isoform usage between individuals in separately sampled sets of individuals from southern (12 samples) or northern (8 samples) populations, respectively. This is in general agreement with an analysis of EST and cDNA data that estimated that 21% of human AS genes are affected by polymorphisms that alter the relative abundances of alternative isoforms [41], and an RNA-Seq analysis of different tissues in 6 human individuals, which estimated that between 10% and 30% AS events show individual-specific variation [1]. Most previous AS studies have concentrated on variation in splicing pattern across tissues, which is probably controlled by the availability of trans-acting factors in different cell types. On the other hand, where it has been studied, genotype-specific variation in splicing is caused by cis-acting SNPs affecting the splice-site region or splicing regulatory sequences [45,46]. Thus, the high degree of SNP polymorphism in wild populations of *P. trichocarpa*[24,25] may contribute to the diversity of AS variants among different *P. trichocarpa* genotypes.

Interestingly, samples taken from individuals in northern populations had more variation between individuals (13%) than samples taken from southern population individuals (7%). We have observed that *P. trichocarpa* individuals from northern populations exhibit higher nucleotide diversity than those from southern populations (A. Geraldes, C. Douglas, Q. Cronk, unpublished). Thus, higher SNP diversity could contribute higher AS variation in the xylem collected from north population individuals. However, it is possible that some of the difference in levels of AS variation between the separately sampled southern and northern populations is the result of environmental rather than genotypic effects on AS variation, and further work on greater numbers of RNA-Seq samples isolated at similar times from trees grown in common gardens, will be necessary to compare the AS differences between southern and northern populations.

High-throughput transcriptome studies are producing a fast-growing catalog of splicing variation in human populations, but so far information on the functional impact of such splicing variation is limited, and few if any analogous studies are available for plants. Thus, our results provide a new glimpse of the evolutionary and phenotypic consequences of AS variation in plant populations. If the AS events conserved in 20 individuals were functionally relevant, they would be expected to be under strong purifying selection and would thus be expected to have significant impacts on phenotype, which will need to be tested by functional studies of selected examples (e.g. [43]). However, we also found a large number of putative genotype-specific AS events at apparent low frequency levels in the population. Some variants may be rare because they have recently arisen, or because they are deleterious and are being selected against, but it is also possible that some variants play a role in adaptation and may be present in at a certain frequency in different populations of the species. The latter possibility can be tested by larger scale population-wide transcriptome studies. Nevertheless, they suggest that, like SNP variants in a relatively small number of genes important for adaptation in *P. trichocarpa*[47], some of the AS variation in wild populations of *P. trichocarpa* could contribute to phenotypic variation important for local adaptation. However, many of these low frequency variants may represent neutral variation. Thus, AS variation may provide a reservoir of novel exons into a minority (minor-isoform) of a gene’s transcripts as suggested by Modrek and Lee [48]. Because a gene’s ancestral function would be maintained by the major-isoform, this may free the minor-isoform from functional constraint, thus reducing purifying selection. Thus, new exons/introns appearing initially as minor splicing isoforms, may gradually gain functions over time, and became constitutive exons correlated with mutations that creating stronger splice sites.

## Conclusions

In summary, this genome-wide analysis of alternative splicing reveals high levels of AS in *P. trichocarpa*, and shows that splicing differences between individuals, including quantitative differences in isoform ratios, are frequent in *P. trichocarpa*. This suggests the hypothesis that individual-specific alternative splicing is a mechanism that accounts for part of individual phenotypic variation in the plant populations, and several avenues are open to testing of this hypothesis in the future. Future studies are needed to identify and elucidate the detailed molecular mechanisms underlying potential splicing regulatory SNPs and trans-factors, as well as to assess the potential functional consequences of genotype-specific alternative splicing events. Furthermore, while the genotypes we sampled from across much of the range of *P. trichocarpa* were grown in a common environment, the extent of subtle environmental variation on expression and splicing patterns within a single genotype remains to be explored, and will be a focus of future studies. Our RNA-Seq data also allowed us to map the transcriptional landscape of the *P. trichocarpa* genome dedicated to the important process of wood formation, and the expression profiles of wood-formation related genes offer opportunities for functional studies of novel wood-related genes in the future.

## Methods

### Sample collection and transcriptome sequencing

The trees sampled in this study with provenances ranging from 59.6°N to 44.0°N (Additional file 1) have been previously described [27] and were maintained in a common garden at the University of British Columbia. Developing xylem from twenty individuals selected for xylem transcriptome analysis [24] was harvested from current year vigorously growing coppice stems the first week of July, 2008 (samples PT02-PT13) or the first week of July, 2009 (samples PT14-PT21) at mid-day, bark removed, and developing secondary xylem tissue scraped using razor blades. The tissue was immediately frozen in liquid nitrogen and stored at −80°C until used for RNA extraction. Library preparation and Illumina GAII sequencing were carried out as described [24].

### Analysis of alternative splicing by real-time PCR

Total RNAs were isolated from developing xylem tissue, using PureLink RNA reagent (Invitrogen, USA), and cleanup and purified with Qiagen RNeasy plant mini kit (QIAGEN, USA) according to the manufacturer’s instructions as previously described [24]. RNA was the same set of samples used for Illumina GAII sequencing analysis. 2 μg of total RNA was used for reverse transcriptase synthesis using the Omniscript RT Kit (Qiagen, USA). Gene-specific PCR primers were designed spanning the location containing alternative splicing events. Primer pairs for POPTR_0001s00260 are 5′ ATGTCACTCTCCTTGCAATC and 3′ TCAGGCACGATCTCACTCA. Primer pairs for POPTR_0002s21650 are 5′ CCAGAGGACATGACCACATC and 3′ CTATGAAGATTTCCAAAACCA. cDNA amplification using locus-specific primer pairs was done with Quick-Load *Taq* 2X Master Mix (New England Biolabs) using PCR condition at initial denaturation at 95°C for 30 seconds, 40 cycles of 95°C for 30 sec, 55°C for 30 sec, 68°C for 1.5 minute, and extension at 68°C for 10 minutes.

### Mapping short reads to the *P. trichocarpa* genome

Illumina GAII RNA-Seq reads [24] were aligned to the *P. trichocarpa* genome v. 2.2 (http://jgi-psf.org/pub/compgen/phytozome/v6.0/Ptrichocarpa/assembly/) using SOAP (v2.19) software [29]. The mapping criteria were as follows: matches should be collinear to the genome allowing up to three mismatches, but no indels. SOAPsplice (v1.1) was used for genome-wide *ab initio* detection of splice junction sites from RNA-Seq [30]. SOAPsplice is an effective tool for detecting not only known splice junctions but also novel junctions. All reads that could not be matched intact on the genomic sequence were searched to find a junction alignment. We required a junction site to be supported by at least two reads with non-repetitive match position and also to have a minimum of four bases on both sides of the junction.

### Identification and quantification of alternative splicing using RNA-Seq

A gene was determined as alternatively spliced if there was evidence of a 5′splice site (SS) spliced to multiple 3′SS, or of a 3′SS spliced to multiple 5′SS, or of at least 1X coverage across full length of one intron (supported by junction reads). We empirically classified the AS events into four different types according to the structures of exons. These four types include intron retention (IR), alternative 5′ splice site (A5SS), alternative 3′ splice site (A3SS) and exon skipping (ES), as described by Wang [1] and Zhang [11]. For each of AS event, reads deriving from specific regions can support the expression of one alternative isoform or the other. For example of an A3SS event, if 12 splice junctions reads mapped to a splice junction joining a 5′SS to a 3′SS, and 4 splice junction reads mapped to a splice junction joining the same 5′SS to a different 3′SS, the first 3′SS would be considered Long (L)-isoform (inclusion), with a “Isoform(L) frequency” of 12 / (12+4) = 75%, as described by Wang [1].

### Normalization of gene expression levels based on RNA-Seq

Gene expression levels based on RNA-Seq data were measured as numbers of fragments per kilobase of exon in a gene per million fragments mapped (FPKM) [32]. Mean read density values for exons, introns, and intergenic regions were computed in units of fragments per kilobase of exon [intron/intergenic] model per million mapped reads, as described by Wang [1].

### Alternative splicing variation analysis

To assess the degree of similarity between different individuals, we performed pairwise comparisons between every sample pair, correlating the isoform ratios of AS events. To exclude low abundance transcripts that could represent “transcriptional noise” of biological or technical origin, the significance of which is under debate [33], we filtered the data to include only splice variants of genes with FPKM values of ≥ 5 in all individuals. Spearman correlation coefficients were computed for each pairwise comparison, and the resulting correlation matrix was clustered using average linkage hierarchical clustering to generate a tree (Figure 5A). To assess possible individual-specific expression for each event, a Fisher’s exact test was performed to evaluate the significance of the 2x2 table in which reads were divided by: 1) individual of origin (i.e. PT02 versus PT03); and 2) read type (i.e. long isoform versus short isoform). Variation in AS events at an adjusted Fisher’s exact *P-*value cutoff corresponding to a false discovery rate of 5% (in the Benjamini-Hochberg sense) between at least one pair of individuals within the population set were considered significant individual-specific expression.

## Availability of supporting data

All sequences were deposited in the Short Read Archive at NCBI under accession number SRA026096. All expression and AS event data are found in Additional file 4 and Additional file 5.

## Competing interests

The authors declare that they have no competing interests.

## Authors’ contributions

HB: Performed bioinformatic analyses, analyzed data, wrote manuscript. EL: Performed experiments. SDM: Conceived of study, contributed to data generation, reviewed manuscript. QCBC: Conceived of study, contributed to data generation, reviewed manuscript. YAEK: Conceived of study, supervised bioinformatics work, reviewed manuscript. CJD: Conceived of study, helped generate data, analyzed data, supervised bioinformatics and experimental work, wrote manuscript. All authors read and approved the final manuscript.

## Supplementary Material

Additional file 1 Origin of 20 individual samples. Click here for file

Additional file 2 Summary of mRNA-Seq read counts and mapping statistics. Click here for file

Additional file 3 Number of splice junctions in 20 individuals. Click here for file

Additional file 4 Top 100 xylem expressed genes and xylem FPKM values for all v. 2.2 genes in 20 individuals. Click here for file

Additional file 5 List of AS events in 20 individuals. Click here for file

Additional file 6 Cell wall formation and TF related AS genes and events. Click here for file
