# Supplementary material for: The developing xylem transcriptome and genome-wide analysis of alternative splicing in Populus trichocarpa (black cottonwood) populations
Source: BMC Genomics. 2013 May 29;14:359. doi: 10.1186/1471-2164-14-359 (PMC3680236; doi:10.1186/1471-2164-14-359)
Supplement: Additional file 1 — Origin of 20 individual samples. [file 1471-2164-14-359-S1.pdf]

Additional file 1 - Origin of individual samples

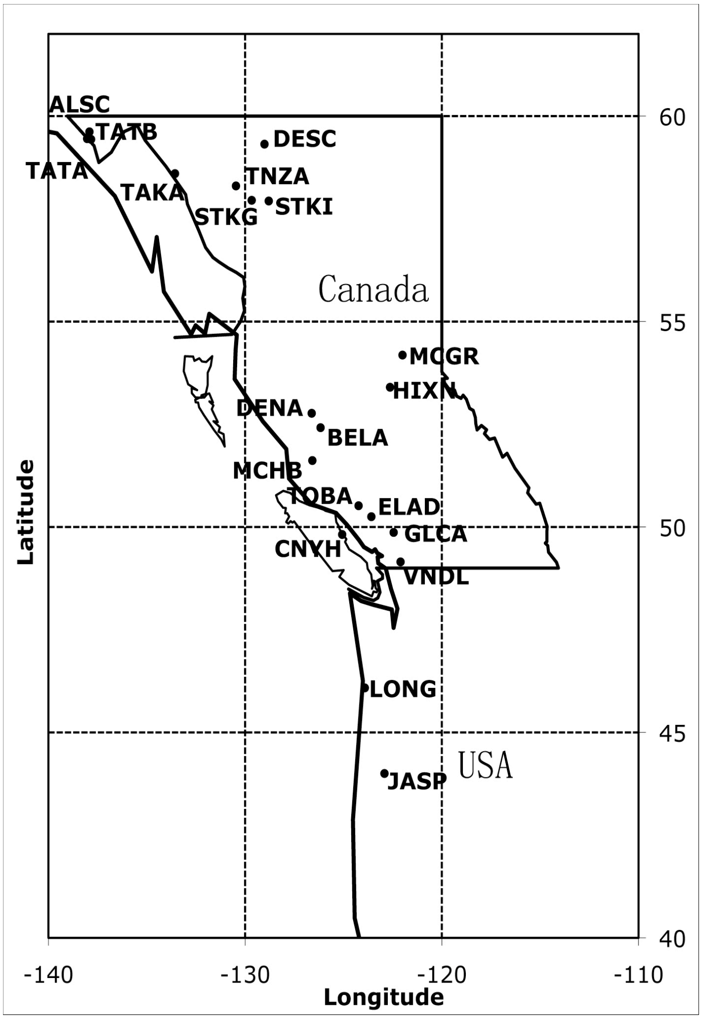

Individual	Code	Longitude	Latitude
DENA	PT0002	-126.62	52.77
BELA	PT0003	-126.17	52.42
CNYH	PT0004	-125.07	49.82
VNDL	PT0005	-122.10	49.15
MCGR	PT0006	-122.00	54.18
HIXN	PT0007	-122.63	53.40
JASP	PT0008	-122.92	44.00
LONG	PT0009	-123.92	46.08
GLCA	PT0010	-122.45	49.87
ELAD	PT0011	-123.58	50.25
MCHB	PT0012	-126.58	51.62
TOBA	PT0013	-124.23	50.52
ALSC	PT0014	-137.92	59.62
TAKA	PT0015	-133.57	58.60
DESC	PT0016	-129.02	59.32
STKI	PT0017	-128.8	57.93
TATA	PT0018	-138.03	59.45
TNZA	PT0019	-130.47	58.30
TATB	PT0020	-137.83	59.43
STKG	PT0021	-129.67	57.95
