# Supplementary material for: The developing xylem transcriptome and genome-wide analysis of alternative splicing in Populus trichocarpa (black cottonwood) populations
Source: BMC Genomics. 2013 May 29;14:359. doi: 10.1186/1471-2164-14-359 (PMC3680236; doi:10.1186/1471-2164-14-359)
Supplement: Additional file 2 — Summary of mRNA-Seq read counts and mapping statistics. [file 1471-2164-14-359-S2.pdf]

**Additional file 2. Summary of mRNA-Seq read counts and mapping statistics**

| | | Genomic Reads | | Junction Reads |
|---|---|---|---|---|
| Individuals | NO. Reads | unique | non-unique | unique |
| PT02 | 58.9M | 39.5M | 4.9M | 5.5M |
| PT03 | 51.2M | 35.1M | 4.7M | 4.3M |
| PT04 | 66.5M | 45.4M | 7.3M | 6.3M |
| PT05 | 61.3M | 41.5M | 8.1M | 6.0M |
| PT06 | 59.9M | 40.8M | 5.8M | 5.1M |
| PT07 | 60.0M | 38.5M | 5.2M | 4.3M |
| PT08 | 52.1M | 36.4M | 4.2M | 5.0M |
| PT09 | 62.8M | 41.4M | 6.1M | 5.2M |
| PT10 | 90.4M | 58.6M | 9.4M | 8.5M |
| PT11 | 66.7M | 28.2M | 6.5M | 4.0M |
| PT12 | 106.4M | 66.5M | 10.5M | 9.5M |
| PT13 | 121.8M | 78.8M | 13.4M | 12.0M |
| PT14 | 96.9M | 60.3M | 9.9M | 7.8M |
| PT15 | 110.5M | 70.2M | 11.1M | 9.8M |
| PT16 | 98.0M | 65.0M | 10.6M | 9.0M |
| PT17 | 100.1M | 65.8M | 9.7M | 9.3M |
| PT18 | 94.8M | 61.0M | 9.3M | 8.2M |
| PT19 | 100.3M | 65.0M | 10.1M | 9.2M |
| PT20 | 106.4M | 62.9M | 10.5M | 8.6M |
| PT21 | 102.5M | 60.0M | 10.2M | 8.1M |
| Total | 1668M | 1061M | 169M | 146M |
| Fractions | 100% | 63.6% | 10.1% | 8.7% |
