# Supplementary material for: The developing xylem transcriptome and genome-wide analysis of alternative splicing in Populus trichocarpa (black cottonwood) populations
Source: BMC Genomics. 2013 May 29;14:359. doi: 10.1186/1471-2164-14-359 (PMC3680236; doi:10.1186/1471-2164-14-359)
Supplement: Additional file 3 — Number of splice junctions in 20 individuals. [file 1471-2164-14-359-S3.pdf]

**Additional file 3. Number of splice junctions**

| Individuals | Known junctions | New junctions |
|---|---|---|
| PT02 | 95049 | 15873 |
| PT03 | 93976 | 8677 |
| PT04 | 96862 | 15668 |
| PT05 | 96741 | 13589 |
| PT06 | 93395 | 11479 |
| PT07 | 93225 | 12071 |
| PT08 | 97164 | 14172 |
| PT09 | 97483 | 14051 |
| PT10 | 99039 | 17163 |
| PT11 | 89840 | 5994 |
| PT12 | 97636 | 15686 |
| PT13 | 103213 | 22913 |
| PT14 | 98243 | 19328 |
| PT15 | 103719 | 24157 |
| PT16 | 104080 | 22075 |
| PT17 | 101362 | 7326 |
| PT18 | 92123 | 27624 |
| PT19 | 98345 | 7045 |
| PT20 | 92537 | 18465 |
| PT21 | 98114 | 18020 |
| Total | 118751 | 80345 |
